# Supplementary material for: Revised Hammersmith Scale for spinal muscular atrophy: Inter and intra-rater reliability and agreement
Source: PLoS One. 2022 Dec 21;17(12):e0278996. doi: 10.1371/journal.pone.0278996 (PMC9770369; doi:10.1371/journal.pone.0278996)
Supplement: Attachment — Submitted filename: Response to reviewers.pdf [file pone.0278996.s002.pdf]

The Dubowitz Neuromuscular Centre
UCL Great Ormond Street Institute of Child Health

Joseph Donlan
Editorial Office PLOS ONE

5th August 2022

Dear Mr Donlan,

We thank the reviewer for the careful reading and constructive criticism of our manuscript.

Please find attached our first revision of the manuscript PONE-D-21-36288 "*Revised Hammersmith Scale for Spinal Muscular Atrophy: inter and intra-rater reliability and agreement*" for further consideration for publication in PLOS ONE. We have dealt with the helpful reviewer's comments and feel the revisions as a result make this paper stronger. We have addressed each point related to journal requirements and the reviewer's comments, as outlined below in this letter and, as requested, attach an updated version of the manuscript, a tracked changes version of the manuscript, new figures 1, 2 and 3, and a supplementary minimal dataset which we consider would now meet your required specifications.

We hope this revised version will now be acceptable for publication in the PLOS ONE. If you require any additional information, please do not hesitate to contact us.

Yours sincerely,

Danielle Ramsey
Honorary Research Physiotherapist
Dubowitz Neuromuscular Centre, UCL GOS Institute of Child Health, London, UK

Senior Lecturer & Course Leader for Physiotherapy
School of Health & Sports Science, University of Suffolk, Ipswich, UK

Rebuttal Response to Reviewers

References to page and line number in the author response to the reviewer's comments detailed throughout the rebuttal comments relate to the 'Revised Manuscript with Track Changes' document.

Response to Journal Requirements:
When submitting your revision, we need you to address these additional requirements.

Journal Requirements:	Response:
1. Please ensure that your manuscript meets PLOS ONE's style requirements, including those for file naming.	We have adhered to the PLOS ONE style requirements for the revised manuscript and associated documents. We have formatted the affiliations and current address for the first author. We have indented each paragraph as in the manuscript formatting guidelines, and have formatted the references section and named files appropriately. Please see the tracked changes document.
2. You indicated that you had ethical approval for your study. In your Methods section, please ensure you have also stated whether you obtained consent from parents or guardians of the minors included in the study or whether the research ethics committee or IRB specifically waived the need for their consent.	We have amended the manuscript accordingly (P8 L185-7 and P10-11 L240-2) and provided more detail with regards who informed consent was obtained from, in addition we have amended the ethics statement in the submission form:<br><br>"The study protocol was given a favourable ethical opinion by the UCL Research Ethics Committee (REC) on 11/05/2015 REC reference 6639/001 subsequent amendments were approved by the committee on 08/06/2015 and 14/08/2015. Research & Development approval was granted from Great Ormond Street Hospital joint research office to conduct this study with NHS staff. Local research and development approval from North Star Clinical Network NHS sites was sought to include their staff in this study. All raters participating in the study gave their written informed consent to participate. This study was affiliated to the longitudinal observational cohort study SMA REACH UK. SMA REACH UK was granted ethical approval (London – Bromley REC reference 13/LO/1748) to record assessment videos of participants, whom had given their informed consent for training purposes (for minors this included parents/guardians informed consent together with minors giving their informed assent). To use these videos in this reliability study a substantial amendment for SMA REACH UK 13/LO/1748 was submitted to the Bromley NHS REC on 28/01/2015 and was granted favourable opinion on 05/02/2015."
3. We note that the grant information you provided in the 'Funding Information' and 'Financial Disclosure' sections do not match.<br>When you resubmit, please ensure that you provide the correct grant numbers for the awards you received for your study in the 'Funding Information' section. The financial	We have updated the funding information to reflect the funding associated with the time this study was undertaken and to clarfy current funding, please see below.

Journal Requirements:	Response:
disclosure section has now been updated, please see response to journal requirements point 4.	"This study was supported, in the UK, by the SMA REACH UK project (www.smareachuk.org). FM is the chief investigator of the SMA REACH project. At the time of the study funding of the SMA REACH UK Project was provided by the SMA Trust and Muscular Dystrophy UK (07DN02; 37787 http://www.musculardystrophyuk.org/grants/clinical-trial-coordinators/), the MRC Translational Research Centre at UCL and Newcastle (MR/K501074/1), and the National Institute for Health Research Biomedical Research Centre (515048) at Great Ormond Street Hospital for Children NHS Foundation Trust and University College London (http://www.gosh.nhs.uk/research-and-innovation/nihr-great-ormond-street-brc/about-brc). This funding supported the SMA REACH UK Project and the positions of FM, MS, DR (and AM in Newcastle), and included support for hosting international workshops. Commercial funding for the SMA REACH UK project has been provided by Biogen Inc since December 2017 (Grant Number: 13/LO/1748, IRAS project ID: 122521), via UCL and GOSH. Both historic and current funders had no role in study design, data collection and analysis, decision to publish, or preparation of the manuscript."
4. Thank you for stating the following in the Financial Disclosure section: "This study was supported, in the UK, by the SMA REACH UK project (www.smareachuk.org). FM is the chief investigator of the SMA Reach project. Commercial funding for the SMA REACH UK project is provided by Biogen Inc (REC reference: 13/LO/1748, IRAS project ID: 122521), via UCL and GOSH. Historically, funding of the SMA REACH UK Project has also been provided by the SMA Trust and Muscular Dystrophy UK (07DN02; 37787 http://www.musculardystrophyuk.org/grants/clinical-trial-coordinators/), the MRC Translational Research Centre at UCL and Newcastle (MR/K501074/1), and the National Institute for Health Research Biomedical Research Centre (515048) at Great Ormond Street Hospital for Children NHS Foundation Trust and University College London (http://www.gosh.nhs.uk/research-and-innovation/nihr-great-ormond-street-brc/about-brc). The funders had no role in study design, data collection and analysis, decision to publish, or preparation of the manuscript."<br><br>We note that you received funding from a commercial source: Biogen Inc<br>Please provide an amended Competing Interests Statement that explicitly states this commercial funder, along with any other relevant declarations relating to employment, consultancy, patents, products in development, marketed products, etc.<br><br>Within this Competing Interests Statement, please confirm that this does not alter your adherence to all PLOS ONE policies on sharing data and materials by including the following statement: "This does not alter our adherence to PLOS ONE policies on	As noted in the previous journal requirements point 3 we have updated the financial disclosure to provider further clarity on the historic and current funding for this study. To address journal requirements point 4 we have amended the competing interest information as stated over the page:<br><br>“The SMA REACH UK project has been commercially funded by Biogen Inc since December 2017 (Grant Number: 13/LO/1748, IRAS project ID: 122521), via UCL and GOSH. The funding from Biogen Inc supports the project and employment for the project including Principal Investigator (FM) time, SMA REACH UK trial project manager, data managers, study co-ordinator, and biobank. MS reports participation to Scientific Advisory boards and teaching initiatives for Biogen Inc. The historical and current funding of the SMA REACH UK project does not alter our adherence to PLOS ONE policies on sharing data and materials. If there are restrictions on sharing of data and/or materials, please state these, sharing of data from database upon patient consent.<br><br>DR reports participation in teaching initiatives for Roche.<br><br>GR reports participation in consultancy for Orphazyme.

Journal Requirements:	Response:
sharing data and materials." (as detailed online in our guide for authors http://journals.plos.org/plosone/s/competing-interests). If there are restrictions on sharing of data and/or materials, please state these. Please note that we cannot proceed with consideration of your article until this information has been declared.<br><br>Please include your amended Competing Interests Statement within your cover letter. We will change the online submission form on your behalf.	MS reports participation to Scientific Advisory boards and teaching initiatives for Avexis, Biogen, Roche; she is involved as an investigator in clinical trials from Avexis, Biogen and Roche.<br><br>FM reports participation to Scientific Advisory boards and teaching initiatives for Avexis, Biogen, Roche and Novartis. He is a member of the Rare Disease Scientific Advisory Board for Pfizer. He is involved as an investigator in clinical trials from Avexis, Biogen and Roche. In addition he is the principal investigator of the SMA REACH UK clinical network, partially funded by Biogen and SMA UK."
5. In your Data Availability statement, you have not specified where the minimal data set underlying the results described in your manuscript can be found. PLOS defines a study's minimal data set as the underlying data used to reach the conclusions drawn in the manuscript and any additional data required to replicate the reported study findings in their entirety. All PLOS journals require that the minimal data set be made fully available. For more information about our data policy, please see http://journals.plos.org/plosone/s/data-availability.<br><br>Upon re-submitting your revised manuscript, please upload your study's minimal underlying data set as either Supporting Information files or to a stable, public repository and include the relevant URLs, DOIs, or accession numbers within your revised cover letter. For a list of acceptable repositories, please see http://journals.plos.org/plosone/s/data-availability#loc-recommended-repositories. Any potentially identifying patient information must be fully anonymized.<br><br>Important: If there are ethical or legal restrictions to sharing your data publicly, please explain these restrictions in detail. Please see our guidelines for more information on what we consider unacceptable restrictions to publicly sharing data: http://journals.plos.org/plosone/s/data-availability#loc-unacceptable-data-access-restrictions. Note that it is not acceptable for the authors to be the sole named individuals responsible for ensuring data access.<br><br>We will update your Data Availability statement to reflect the information you provide in your cover letter.	We have included the minimal dataset underpinning the results of this study in the supporting information S1 Dataset. This does not include detail regarding the demographics of the raters included in the study, this information, their affiliation with the SMA REACH UK sites and small population studied would mean even anonymised data would have several indirect identifiers which may risk breaking anonymity. Minimal information pertaining to the raters can be found within the participants section within results (P11-12 L255-287). The minimal dataset can also be found within the results section in tables 1, 2 and 3 and within Figures 1, 2 and 3.<br><br>Data beyond the minimal dataset required for this study are not publicly accessible due to it containing sensitive data pertaining to human research participants and may compromise anonymity. However, external parties can request data in aggregate form from the SMA REACH UK steering committee (contact via FM) for researchers who meet the criteria for access to confidential data.<br><br>The supplementary study minimal dataset can be found P24 L539--542:<br>"**Supporting information**<br>S1 Dataset. Study Minimal Dataset. This file contains the minimal dataset underpinning the results for this study. Demographic data is not included in this minimal dataset due to the risk of indirectly identifying participants."
6. Please note that in order to use the direct billing option the corresponding author must be affiliated with the chosen institute. Please either amend your manuscript to change the affiliation or corresponding author, or email us at plosone@plos.org with a request to remove this option.	The corresponding author is affiliated with Univerrsity College London (UCL). We have amended the author's affiliations (P1 L7-8) and made the other author's affiliations clearer also (P1 L7-17).

Journal Requirements:	Response:
7. Please upload a new copy of Figures 1-4 as the detail is not clear. Please follow the link for more information: https://blogs.plos.org/plos/2019/06/looking-good-tips-for-creating-your-plos-figures-graphics/ " https://blogs.plos.org/plos/2019/06/looking-good-tips-for-creating-your-plos-figures-graphics/	The original Figure 1 has been removed from the manuscript (please see response to journal requirement 8 below). The current figs 1, 2 and 3 within the manuscript have been uploaded to the PACE tool to ensure they now meet the formatting and quality requirements for publication and have been new copies have been uploaded with this manuscript. Please see Fig1.tif Fig2.tif Fig3.tif
8. We note that Figure 1 in your submission contain copyrighted images. All PLOS content is published under the Creative Commons Attribution License (CC BY 4.0), which means that the manuscript, images, and Supporting Information files will be freely available online, and any third party is permitted to access, download, copy, distribute, and use these materials in any way, even commercially, with proper attribution. For more information, see our copyright guidelines: http://journals.plos.org/plosone/s/licenses-and-copyright.<br><br>We require you to either (1) present written permission from the copyright holder to publish these figures specifically under the CC BY 4.0 license, or (2) remove the figures from your submission.	The original Figure 1 has been removed from the manuscript and not replaced.
9. Please review your reference list to ensure that it is complete and correct. If you have cited papers that have been retracted, please include the rationale for doing so in the manuscript text, or remove these references and replace them with relevant current references. Any changes to the reference list should be mentioned in the rebuttal letter that accompanies your revised manuscript. If you need to cite a retracted article, indicate the article's retracted status in the References list and also include a citation and full reference for the retraction notice.	The reference list has been reviewed to ensure it meets the requirements of the journal. Amendments have been made to the following:<br>P20 L441 – Ref 1 updated<br>P21 L461 – Ref 9 updated<br>P21 L478 – Ref 16 updated<br>P22 L481 – Ref 17 updated<br><br>In addressing the reviewer's comment point 3 regarding supporting evidence for the Bland Altman limits off agreement we have added 4 further references in the manuscript, references 28-31 (P23 L510-520). Therefore the references which also appear after this section have also been re-numbered to correspond with where they appear in the manuscript.

Reviewers Comments to the Author with regards Journal's questions:

Journal's questions:	Reviewer Comments:	Response:
1. Is the manuscript technically sound, and do the data support the conclusions?	Reviewer #1: Yes	We would like to thank the reviewer for their positive feedback.

Journal's questions:	Reviewer Comments:	Response:
The manuscript must describe a technically sound piece of scientific research with data that supports the conclusions. Experiments must have been conducted rigorously, with appropriate controls, replication, and sample sizes. The conclusions must be drawn appropriately based on the data presented.		
2. Has the statistical analysis been performed appropriately and rigorously?	Reviewer #1: Yes	We would like to thank the reviewer for their positive feedback.
3. Have the authors made all data underlying the findings in their manuscript fully available?<br><br>The PLOS Data policy requires authors to make all data underlying the findings described in their manuscript fully available without restriction, with rare exception (please refer to the Data Availability Statement in the manuscript PDF file). The data should be provided as part of the manuscript or its supporting information, or deposited to a public repository. For example, in addition to summary statistics, the data points behind means, medians and variance measures should be available. If there are restrictions on publicly sharing data—e.g. participant privacy or use of data from a third party—those must be specified.	Reviewer #1: Yes	We would like to thank the reviewer for their positive feedback, and we have expanded our access to the minimal dataset underpinning the results as described in the response to point 5 of the journal requirements.
4. Is the manuscript presented in an intelligible fashion and written in standard English?<br><br>PLOS ONE does not copyedit accepted manuscripts, so the language in submitted articles must be clear, correct, and unambiguous. Any typographical or grammatical errors should be corrected at revision, so please note any specific errors here.	Reviewer #1: Yes	We would like to thank the reviewer for their positive feedback.

<u>Reviewers Comments on the Manuscript</u>

Reviewer #1: The authors have been rigorous in assessing the reliability of the HMS. On my part, I find few observations, but these observations may be fundamental. In particular, I think the study may be a good marker for further psychometric studies of the HMS.

Response:
We would like to thank the reviewer for their positive feedback and their constructive suggestions which we believe will make this manuscript stronger. We have responded to systematically as detailed below.

Reviewer #1 Comments on the Manuscript:	Response:
1. The assessment of the reliability of the score first assumes that the unidimensionality of the items included in this score is satisfactory. Even in a small sample, the authors should report evidence of unidimensionality of the	We thank the reviewer for highlighting this omission, and have addressed this by including a section on the Revised Hammersmith Scale in methods to describe the psychometric properties underpinning the scale. The RHS was developed using the Rasch Unidimensional Measurement Model, and this the unidimensionality is reported in relation to the Rasch analysis undertaken in a concurrent study.

Reviewer #1 Comments on the Manuscript:	Response:
instrument, even with "simple" statistics, e.g., inter-item correlation, item-test correlation, etc.	These new section can be found on P5 L96-110:<br>"The scale was developed using modern psychometric techniques and latent measurement theory via the Rasch Unidimensional Measurement Model (unrestricted and simple logistic model) [17, 18]. Rasch analysis identified unidimensionality of the RHS as acceptable with t-test 7.3%, binomial test lower 95% confidence interval proportion, 0.05 [17]."
2. In Table 1, they can also report the standard deviation or other univariate estimator of score variability.	We thank the reviewer for highlighting this omission, and have addressed this by including a column to report the SD for RHS total score in table 1 and also table 2.<br><br>These amendments can be found on P13 L299-300, and P14 L309-312.
3. "...were consulted regarding setting the level 190 of agreement for the Bland Altman analysis [17]. The experts agreed that the limits of agreement (precision) of the anticipated differences between raters/intra-rater for the total score of the test would lie within ±2 points overall and therefore ±2 was the set limits of agreement (precision) for this study..."<br><br>Here it is required to describe what was the rationale for arriving at this magnitude of anticipated differences.	We thank the reviewer for suggesting these amendments and have addressed this by way of further explanation, and reference to scale variance related to the original HFMSE.<br><br>These amendments can be found on P9 L209 to P10 L221:<br>"To investigate the level of agreement (precision) of scale scoring expert physiotherapists were consulted regarding setting the level of agreement for the Bland Altman analysis [17]. Evidenced values for the level of agreement were unavailable at the time of this study due to the RHS being a new scale and entering a pilot phase of testing, therefore properties such as scale variance had not yet been investigated. The experts agreed that the limits of agreement (precision) of the anticipated differences between raters/intra-rater for the total score of the test would lie within ±2 points overall and therefore ±2 was the set limits of agreement (precision) for this study. This level was based upon their expert experience of using the Hammersmith Functional Motor Scale (HFMS), Hammersmith Functional Motor Scale Expanded (HFMSE) and their involvement in developing and testing the RHS. The levels of agreement set by the experts, at the time of this study, were not dissimilar to the scale variance reported in the literature for the HFMS, HFMSE, and Modified HFMS [28-31]."
4. It is advisable to summarily describe the content of the HMS items, perhaps in a section within the Method, e.g., Instrument.	We thank the reviewer for highlighting this omission, and have addressed this by including a section to describe the contents and psychometric properties of the on the Revised Hammersmith Scale in methods.<br><br>These new section can be found on P5 L95-110:<br>"**Revised Hammersmith Scale**<br>The RHS is a clinician rated SMA specific outcome measure containing 36 items which assess physical motor performance [17]. The scale assesses motor functional activities related to sitting, supine, rolling, prone, ability to move and get up from the floor, balance, standing, run/walk, stairs, ascending and descending a step and the ability to jump. Thirty-three items are graded according to an ordinal 0, 1, 2 scale where 0 represents the least physical ability or function achieved, and 2 the highest. Three items are graded 0 and 1 where 0 represents an inability to complete the item, and 1 represents achieving the item. Two timed tests are included within the scale, and WHO motor milestones can also be completed concurrently. The scale was developed using modern psychometric techniques and latent measurement theory via the Rasch Unidimensional Measurement Model (unrestricted and simple logistic model) [17, 18].

Reviewer #1 Comments on the Manuscript:	Response:
	Rasch analysis identified unidimensionality of the RHS as acceptable with t-test 7.3%, binomial test lower 95% confidence interval proportion, 0.05 [17]. Reliability of the RHS was demonstrated to be good with a high Person Separation Index (PSI) of 0.98 [17]. Dependency was seen between items tested on the right and left and rolling supine to prone and prone to supine however removing these items did not alter the PSI [17]."
5. I suggest including graphs of the distribution of scores, e.g., histograms.	We thank the reviewer for this feedback. We have plotted histograms for the results of this study, however, we feel the figs 1, 2 and 3, and tables 1, 2 and 3 within the manuscript demonstrates the distribution of scores. In addition the supplementary information included in the revised manuscript contains the individual rater scores for Survey 1 and Survey 2 enabling greater transparency of the data. Therefore, at this time we have not chosen to incorporate further graphs into this manuscript.<br><br>The supplementary study minimal dataset can be found P24 L539-542:<br>"**Supporting information**<br>S1 Dataset. Study Minimal Dataset. This file contains the minimal dataset underpinning the results for this study. Demographic data is not included in this minimal dataset due to the risk of indirectly identifying participants."
6. The decision to use a two-week time interval may be a bit more applied.	We thank the reviewer for their suggestion and have followed your recommendations by including further explanation related to this point.<br><br>These amendments can be found on P7 L163-5:<br>"Two weeks were chosen to ensure enough time between surveys so that the participants remained blind to their previous scores and to maintain currency and engagement with the study."
7. In this first reliability study, internal consistency should also be reported, for example with coefficient alpha (with confidence intervals).	We thank the reviewer for highlighting this omission, and have addressed this by including a section on the Revised Hammersmith Scale in methods to describe the psychometric properties underpinning the scale. The RHS was developed using the Rasch Unidimensional Measurement Model, and the Rasch analysis equivalent to internal consistency is the concept of dependency. Therefore further information has been added to the manuscript regarding the pscyometric properties of dependency.<br><br>These new section can be found on P5 L107-110:<br>"Reliability of the RHS was demonstrated to be good with a high Person Separation Index (PSI) of 0.98 (17). Dependency was seen between items tested on the right and left and rolling supine to prone and prone to supine however removing these items did not alter the PSI [17]."
